# The clinical and pathological features of Kimura disease in pediatric patients

**DOI:** 10.3389/fmed.2024.1352206

**Published:** 2024-04-25

**Authors:** Lili Liu, Wen Zhang, Minjie Huang, Kaiji Li, Li’ou Zhu, Liqin Ke, Juan Huang, Ying Wu

**Affiliations:** Wuhan Children’s Hospital (Wuhan Maternal and Child Healthcare Hospital), Tongji Medical College, Huazhong University of Science & Technology, Wuhan, China

**Keywords:** pediatric pathology, Kimura disease, IgE, lymphadenopathy, immunohistochemistry

## Abstract

**Background:**

Kimura disease is characterized by inflammation, with its underlying causes remaining uncertain. There is a lack of comprehensive and systematic research on the pathology of this condition in pediatric patients. Our objective is to study the clinical and pathological attributes of Kimura disease in pediatric patients and investigate the potential diagnostic significance of immunoglobulin E (IgE) in this context.

**Methods:**

Clinical and laboratory information, pathological characteristics, and follow-up data were correlated to examine the distinctive features. Immunohistochemistry, acid-fast staining, and molecular assay were used to identify the presence of IgE and pathogens.

**Results:**

We conducted an analysis of five cases of Kimura disease in pediatric patients at our hospital. The patients’ ages ranged from 5 years and 7 months to 14 years and 2 months, with 4 (80%) being male. The most common site was the head and neck region, particularly the postauricular subcutaneous area. Eosinophilia was observed in four patients (80%), and two patients (40%) had elevated serum immunoglobulin E (IgE) levels. Histopathological changes included eosinophilic infiltrates, follicular hyperplasia, and the proliferation of postcapillary venules. Immunohistochemical results supported the reactive nature of the lymphoid process and IgE deposition in the follicle, while no specific pathogens were discovered by special staining. All patients underwent surgical excision, and none experienced recurrence in their original location.

**Conclusion:**

Children with Kimura disease show distinct eosinophilic and IgE alterations in both laboratory findings and pathological features. The application of immunohistochemical staining of IgE could serve as a promising marker for diagnosing Kimura disease.

## 1 Introduction

Kimura disease, also known as eosinophilic hyperplastic lymphogranuloma, is a rare inflammatory condition that affects the lymphoproliferative tissue. The condition primarily affects young men of Asian descent in their early 30s and is characterized by painless enlargements, particularly in the head and neck area (1–3). The accurate identification of the deep-seated subcutaneous lesions is crucial to prevent unnecessary treatment and to facilitate further investigation into their causes, as they are frequently misdiagnosed as neoplasms.

Several individual cases have been recorded in adult patients (3–6), but there is an increasing focus on pediatric patients (7) in recent years. This study investigated the clinical and laboratory data, pathological features, and ongoing observation of five children diagnosed with Kimura disease over a period of 7 years at Wuhan Children’s Hospital, Tongji Medical College, and Huazhong University of Science and Technology. The article highlights the significance of precise pathological diagnosis in guiding suitable treatment.

## 2 Materials and methods

We conducted a retrospective analysis of cases diagnosed as lymph node reactive inflammation at Wuhan Children’s Hospital, Tongji Medical College, Huazhong University of Science and Technology, from January 2016 to December 2023. The study adhered to specific morphologic criteria as outlined in existing literature (8). A total of five cases were included in this study, each accompanied by a comprehensive clinical history, including age, gender, duration of symptoms, anatomical location, size of the mass, laboratory findings, and other relevant presentations. All slides underwent independent review by a minimum of two pathologists with at least 3 years of experience in the field. Furthermore, all cases were subjected to follow-up.

## 3 Results

### 3.1 Clinical data and laboratory examination

A summary of the relevant clinical characteristics is outlined in Table 1. The study involved five pediatric patients, ranging in age from 5 years and 7 months to 14 years and 2 months, with a median age of 9 years old. Five cases were diagnosed with Kimura disease based on histopathological examination. Among these patients, four were male (cases 1, 2, 3, and 4), resulting in a male-to-female ratio of 4:1. Five cases were observed to have painless swellings, prompting them to seek medical attention. The duration of these masses varied among the patients, with symptoms lasting from 3 days (case 5) to 10 years (case 3), while the specific duration for case 4 was not provided. It is noteworthy that all patients were under 10 years old when they noticed their nodules.

**Table 1:** Synopsis of the pertinent clinical features.

Patient No.	Age/Gender	Duration of symptoms	Anatomic location	Size (cm)	Blood eosinophilia	IgE level	Other presentation
1	11 years and 6 months/Male	6 years	Postauricular Neck	3.5 × 2 × 0.7 1.6 × 1.2 × 0.5	Elevated	NA	NA
2	11 years/Male	5 years	Postauricular	L: 3.5 × 1.5 × 0.5 R: 2.5 × 2 × 1	Elevated	NA	Small, pale red papules with pruritus on the lower limbs
3	14 years and 2 months/Male	10 years	Postauricular	2 × 2 × 1.5	Elevated	Elevated	Nephrotic Syndrome
4	6 years/Male	NA	Elbow	2.2 × 1.0 × 0.8	Elevated	Elevated	Itchy papules on the lower limbs
5	5 years and 7 months/Male	3 days	Upper arm	1.5 × 1 × 0.4	Normal	NA	NA

Three patients (cases 1, 2, and 3) presented with a painless enlargement in the head and neck area, while the other cases displayed an enlargement in the left elbow (case 4) and the right upper arm (case 5), respectively. Each patient displayed at least one lesion. Cases 1 and 5 exhibited painless solitary masses as their primary clinical manifestation, devoid of any additional symptoms. Notably, cases 2 and 4 displayed varying degrees of cutaneous manifestations accompanied by masses. Specifically, in case 2, the patient developed small, pale red papules with pruritus on the extensor surfaces of both lower limbs 3 years subsequent to the emergence of bilateral masses as he came for treatment. In case 4, itchy papules appeared on the lower limbs over a year after the onset of a mass on the left elbow, lasting for 4 months before seeking medical intervention. Case 3, on the other hand, did not exhibit any cutaneous symptoms; however, the patient concurrently presented with nephrotic syndrome symptoms upon noticing the mass.

In laboratory analyses, four male patients (cases 1, 2, 3, and 4) exhibited a rise in eosinophils in their peripheral blood. Among these patients, two individuals (cases 3 and 4) demonstrated elevated levels of serum IgE, while this particular test was not conducted for the remaining three patients. Furthermore, assessments were conducted to examine the concentrations of cytomegalovirus (CMV) and Epstein–Barr encoding region (EBER) virus IgG and IgM in the patients. Cases 2 and 3 displayed increased levels of CMV IgG and EBER IgG occasionally.

### 3.2 Pathological findings

#### 3.2.1 Macroscopic

All individuals received surgical excision, and subsequent histopathological analysis verified the presence of Kimura disease. The sizes of the masses varied between 1.5 and 3.5 cm, with an average dimension of 2.3 cm. The nodules displayed a gray-white coloration upon dissection, featuring a clearly defined boundary within the tissue.

#### 3.2.2 Microscopic

**Figure 1:**
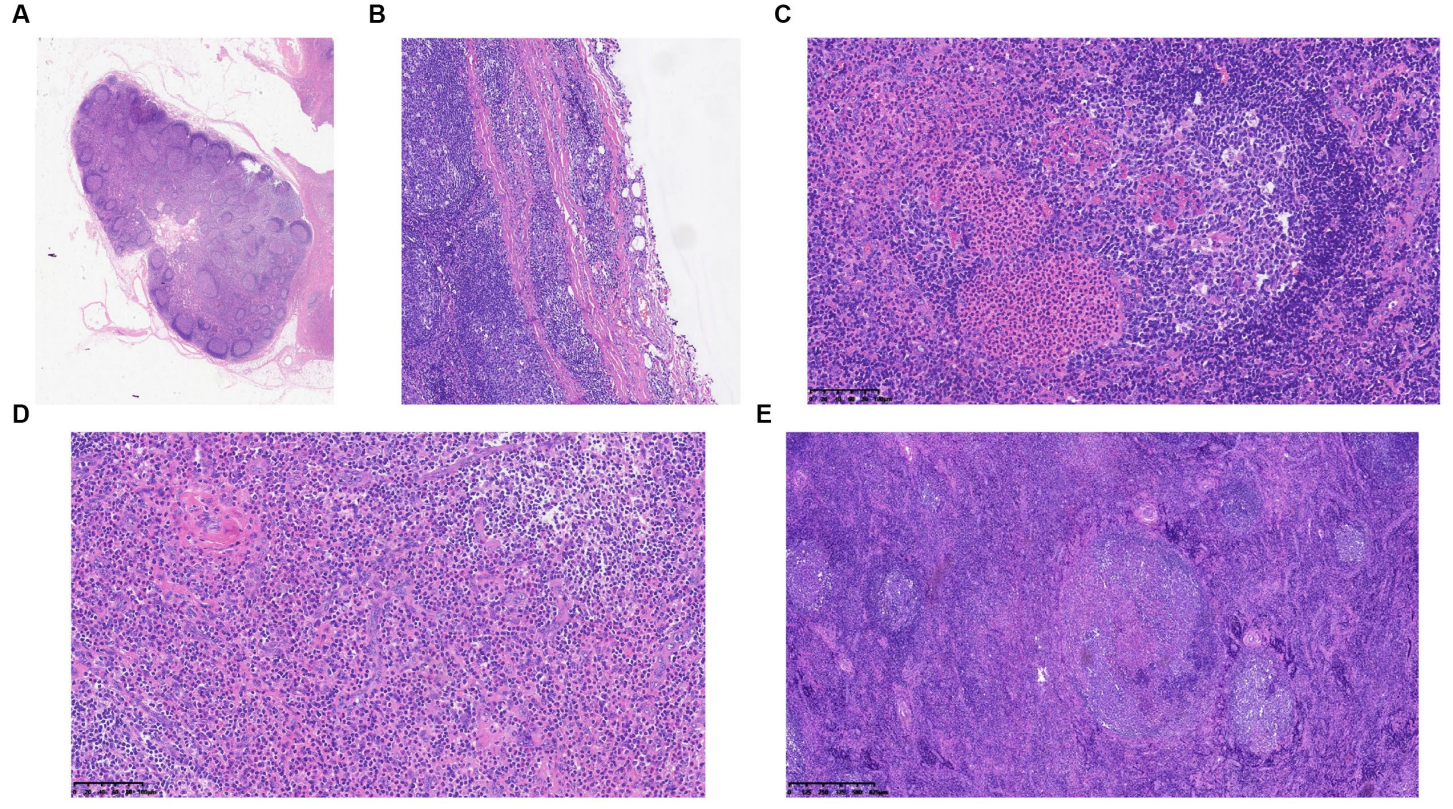
**(A)** Scanning slice shows the intact structure of the lymph node. **(B)** The membrane of the lymph node is thickened and even extended into the nodules. **(C)** Marked eosinophilic infiltration and the characteristic eosinophilic staining fill in the germ center. **(D)** Postcapillary venule proliferation is present in the interfollicular areas. **(E)** The germ centers proliferate within lymph nodes.

The histological characteristics of the five cases exhibit a relatively consistent pattern. When observed under low magnification, the typical structure of the lymph nodes is disrupted, with a notable increase in lymphoid follicles of varying sizes that occupy both the cortex and medulla (Figure 1A). Germinal centers are reserved, and mature lymphocytes infiltrate in it (Figure 1E) in a clustered pattern beneath the capsule, which is thickened and even extended into the nodules (Figure 1B). When viewed under high magnification, a significant number of eosinophils infiltrate the germinal centers and interfollicular areas, leading to the formation of localized eosinophilic abscesses (Figure 1C). In certain regions, there is the presence of homogeneous material that exhibits eosinophilic staining within the germinal centers (Figure 1C). Furthermore, there is evidence of heightened vascularity in the interfollicular areas (Figure 1D).

#### 3.2.3 Special studies

**Figure 2:**
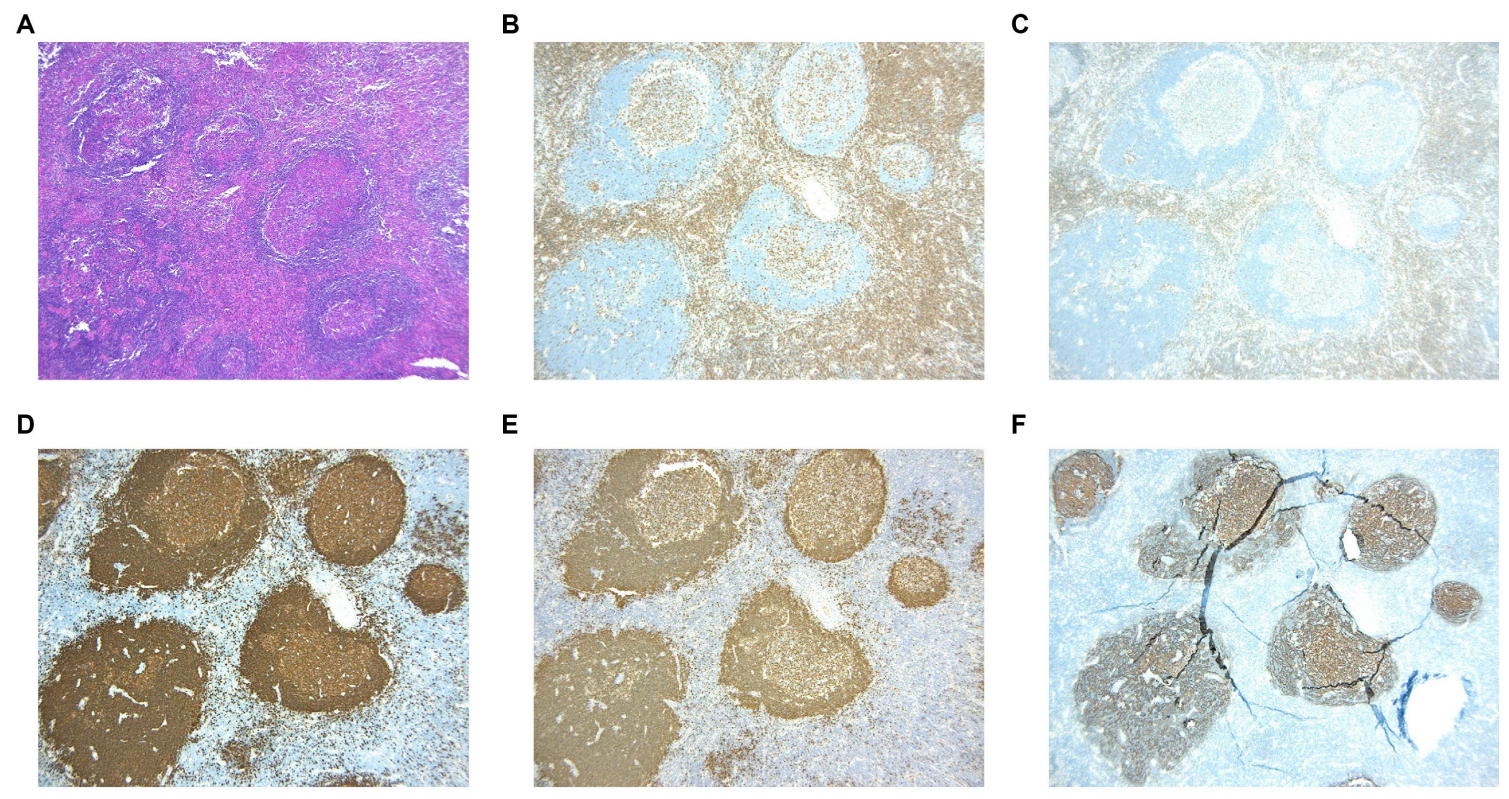
(**A**) HE staining reveals the destruction of lymph node due to the presence of eosinophilic abscess. **(B,C)** Immunohistochemistric staining of CD3 and CD5 marked T cells. **(D,E)** Immunohistochemistric staining of CD20 and PAX5 marked B cells. **(F)** Immunohistochemistric staining of CD21 showed lymphoid follicular.

**Figure 3:**
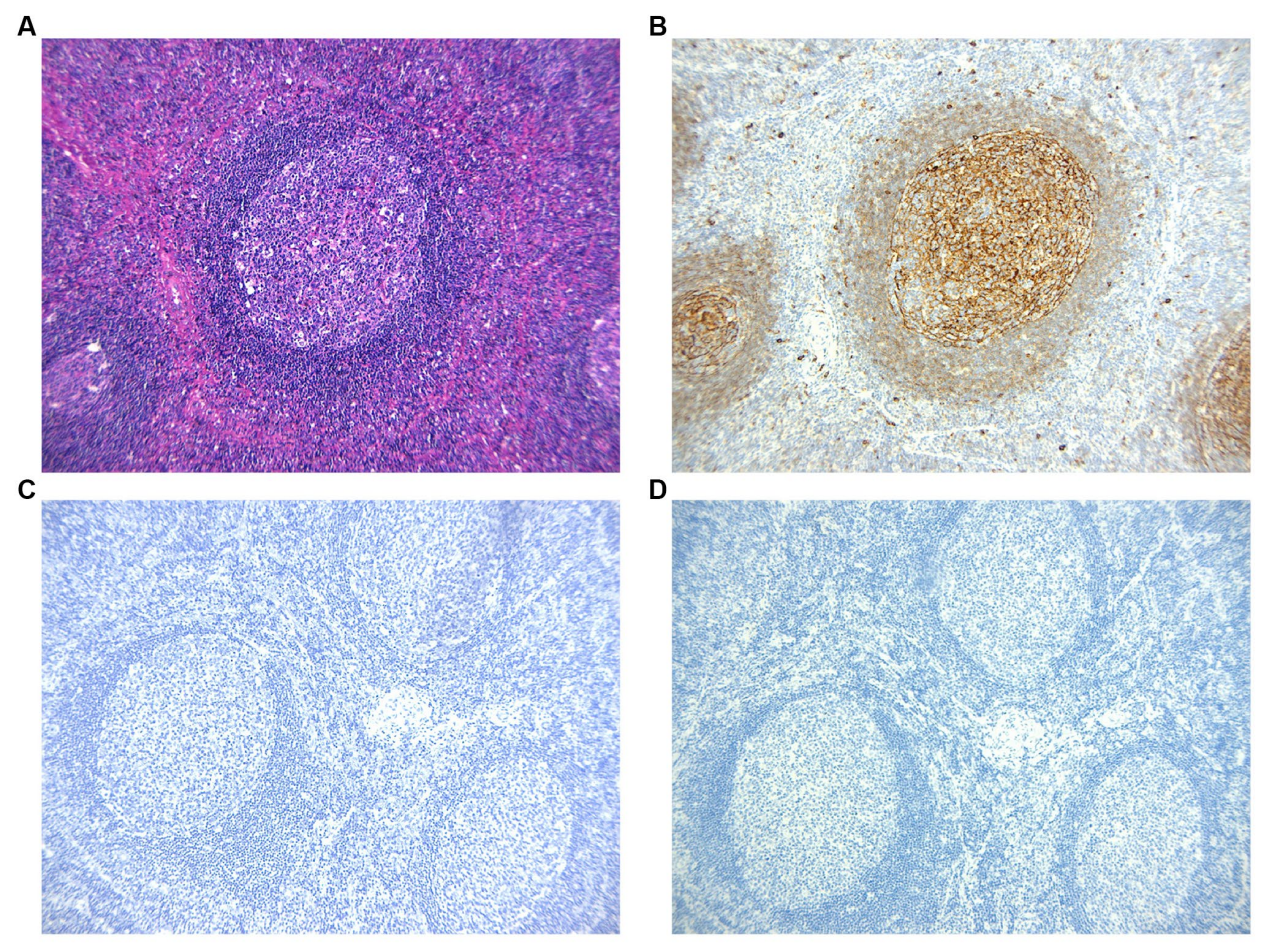
**(A,B)** Immunohistochemistic images show IgE reticular networks in the germinal centers. Non-reactive cells are present in the paracortex. **(C,D)** The immunohistochemical stains of CMV and EBER virus are negative.

The arrangement of T cells (CD3 and CD5) and B cells (CD20 and Pax5) within actively growing follicles in lymph nodes (Figures 2B–E) appeared to be typical, with CD21 staining (Figure 2F) indicating the presence of follicular dendritic networks in germinal centers, local damage caused by eosinophilic abscesses (Figure 2A). Positive germinal center IgE staining (Figures 3A,B) was observed in all cases. The immunohistochemical stain of CMV (Figure 3C) the molecular assay of EBER virus staining (Figure 3D) failed to reveal any relative pathogens, as did the acid-fast stain.

### 3.3 Follow-up data

In December 2023, a follow-up was conducted on the five patients as part of this study. Out of the five patients, one experienced a relapse, while the remaining patients were generally in good health. Cases 1 and 5 achieved complete recovery without further treatment. Case 2, who received steroid treatment for 9 days post-surgery, exhibited a significant reduction in eosinophils to a normal degree and the disappearance of itching symptoms. Case 3 experienced the complete resolution of Kimura disease symptoms after surgery and continued to receive treatment for nephrotic syndrome. Case 4 developed new lesions on the right elbow and one eyelid, which showed intermittent improvement with medication.

## 4 Discussion

Kimura disease, also referred to as eosinophilic hyperplastic lymphogranuloma, is an uncommon lymphoproliferative inflammatory disorder (9). It exhibits a higher prevalence in male individuals compared to female individuals, with a gender ratio of approximately 4.5:1 (7). Due to its gradual onset, the disease often appears without noticeable symptoms, and some individuals tend to disregard it.

The etiology and pathogenesis of Kimura disease remain uncertain. Some researchers have suggested that persistent antigen stimulation following arthropod bites and allergic reactions triggered by parasitic infections such as fungi (10, 11) may influence alterations in immune regulation, potentially contributing to the onset of Kimura disease. In our investigation, the absence of CMV and EBER virus and negative acid-fast staining indicate no association of Kimura disease with these infections. Presently, it is widely accepted that T cells play a crucial modulatory role in eosinophil development, with type 1 and type 2 T helper cells originating from T cells (Th1 and Th2) being the primary factors associated with excessive eosinophil and IgE production (12), thereby precipitating Kimura disease. In our study, four (80%) patients showed elevated eosinophils as well as elevated serum IgE levels in two cases.

Histological examination of stained human tissue samples is considered the gold standard method for diagnosing Kimura disease. Hui et al. (8) provided a comprehensive overview of the histological characteristics of lymph nodes in Kimura disease, including preserved nodal structure, prominent germinal center hyperplasia, eosinophil infiltration, and proliferation of postcapillary venules. These constant features were observed in our five cases. They (8) first suggested the use of immunohistochemistry to demonstrate an IgE reticular network within germinal centers, with the positive expression of IgE observed in 39 out of 44 enlarged lymph nodes from 20 patients with Kimura disease. In line with these findings, IgE was detected in all seven lymph nodes from cases examined in our study. The application of immunohistochemical staining of IgE could serve as a promising approach for diagnosing Kimura disease.

Kimura disease is characterized by the development of painless nodules under the skin, which must be differentiated from other conditions that cause swelling of the lymph nodes. The differential diagnosis typically involves considering lymphoma, lymphadenitis, and lymph node hyperplasia resulting from various causes. Immunohistochemical staining plays a crucial role in distinguishing between lymphoma and reactive lymphadenopathy. Pathologists commonly utilize antibodies such as CD3, CD5, CD20, PAX5, CD21, IgD, and Ki67 for this purpose. The primary considerations for differential diagnosis of lymphadenitis typically encompass tuberculous lymphadenitis, cat scratch disease (CSD), Kikuchi-Fujimoto disease (KFD), and parasitic lymphadenitis. Tuberculous lymphadenitis is characterized by tuberculous nodules containing caseous necrosis surrounded by tissue cells and multinucleated giant cell reactions (13). CSD presents as chronic lymphadenopathy often associated with fever following bites or scratches from animals or plant materials, exhibiting central stellate microabscesses and peripheral granulomatous inflammation histologically (14). KFD is a prevalent lymphadenopathy featuring necrotic nodules comprising numerous tissue cells with round to crescent-shaped nuclei and tissue cell fragments (15), with CD123 immunohistochemical staining aiding in the differentiation of Kikuchi tissue cells (16). Parasitic infections typically manifest as an accumulation of eosinophils forming granulomas, sometimes with observable parasite eggs (17). In pediatric cases, distinguishing Langerhans cell histiocytosis (LCH) is essential, which is characterized by an increase in Langerhans tissue cells and the infiltration of numerous eosinophils. Langerhans cells resembling “coffee beans”-like nuclei are positive for Langerin, S100, and CD1a (18). In each of our five instances, an immunohistochemical stain was conducted on the pediatric individuals to exclude the presence of lymphoma, reactive lymphadenopathy, KFD, and LCH. Following a thorough examination of the patients’ medical records and pertinent special stains, no indications of tuberculosis, CSD, parasitic infection, or LCH were identified.

In addition to the presence of subcutaneous nodules, many individuals diagnosed with Kimura disease also exhibit symptoms such as pruritus, dermatitis, rash, and urticaria, which could potentially be associated with eosinophil infiltration (7). Khoo et al. (19) documented two Chinese adult patients, while Chen et al. (3) described a 13-year-old African American male with a history of eczema. Teraki et al. (20) detailed the case of a 33-year-old man with a childhood history of asthma, who developed pruritic papules on the limbs and trunk 3 years after the emergence of nodules behind the ear. In our investigation, while these five cases did not exhibit signs of eczema, cases 2 and 4 developed pruritic papules on the lower limbs 3 years and 1 year after the onset of subcutaneous nodules, respectively. In case 2, a skin biopsy was performed, revealing the presence of lymphocytes surrounding minor blood vessels. Notably, both patients displayed an elevation in eosinophil levels in their peripheral blood.

In recent years, an increasing number of scholars have found that Kimura disease is often accompanied by kidney disease. Kimura cases with kidney disease can exhibit various pathological changes upon biopsy. These changes include minimal change disease of the kidneys, mesangial proliferative glomerulonephritis, membranous glomerulopathy (21–23), and focal segmental glomerulosclerosis (22). Yamada et al. (24) summarized 175 cases of Japanese Kimura disease patients, among whom 13 clinically presented with nephrotic syndrome. Takei et al. (25) confirmed that elevated serum IgE levels increase the risk of recurrence of minimal change disease. In this study, all cases showed strong expression of IgE in the germinal center, and cases 3 and 4 had elevated IgE levels in serum. Case 3 was accompanied by nephrotic syndrome, and biopsy suggested mesangial proliferative glomerulonephritis. We speculate that IgE is involved in the development of Kimura disease, which may be a concomitant symptom of mesangial proliferative glomerulonephritis in case 3. As the symptoms of Kimura disease completely disappeared and IgE levels returned to normal levels after surgery, the patient continued to receive treatment for nephrotic syndrome.

The typical course of action involves the excision of the masses through surgery, the administration of corticosteroids and biologics, radiotherapy, and immunosuppressive agents. Although surgical resection is the most commonly utilized treatment method, it is also associated with a higher risk of recurrence (26). In our study, cases 1 and 5 did not experience a relapse post-surgery without any additional interventions, whereas case 4, who solely underwent surgery, experienced a relapse on the right elbow and one eyelid. Corticosteroid therapy has proven to be an effective approach to managing the condition. Case 3, who had the concomitant nephrotic syndrome, observed changes in nodule size with intermittent steroid therapy prior to surgery, and case 2, who received a 9-day steroid regimen post-surgery, exhibited a notable decrease in eosinophils without recurrence.

## 5 Conclusion

Kimura disease is a chronic inflammatory condition affecting lymph nodes, characterized by prolonged duration. It is more prevalent in male pediatric patients compared to female pediatric patients. The primary symptom is the painless swelling of lymph nodes. Some individuals may also exhibit a pruritic rash or nephrotic syndrome. Elevated levels of serum eosinophils and IgE, as well as substantial eosinophil infiltration and IgE deposition in germinal centers on histological examination, prove the significant involvement of eosinophils and IgE in the pathogenesis of Kimura disease. The presence of IgE antibodies can aid in distinguishing Kimura disease from other lymphoproliferative disorders. The recurrence rate following surgical intervention in pediatric patients is low, with a favorable prognosis. Combining surgical treatment with oral corticosteroids has been shown to effectively reduce the likelihood of recurrence.

## Data availability statement

The original contributions presented in the study are included in the article/supplementary material, further inquiries can be directed to the corresponding author.

## Ethics statement

The studies involving humans were approved by the Medical Ethics Committee of the Wuhan Children’s Hospital (Wuhan Maternal and Child Healthcare Hospital), Tongji Medical College, Huazhong University of Science and Technology. The studies were conducted in accordance with the local legislation and institutional requirements. The human samples used in this study were acquired from a by-product of routine care or industry. Written informed consent for participation was not required from the participants or the participants’ legal guardians/next of kin in accordance with the national legislation and institutional requirements.

## Author contributions

LL: Writing – review & editing, Investigation, Data curation. WZ: Writing – original draft, Validation, Resources. MH: Writing – original draft, Methodology. KL: Writing – original draft, Methodology. LZ: Writing – original draft, Investigation. LK: Writing – original draft, Investigation. JH: Writing – original draft, Investigation. YW: Writing – review & editing, Supervision, Data curation.
